# Points in Prescribing

**Published:** 1907-06-01

**Authors:** D. M. Macdonald

**Affiliations:** Dunkeld


### The General Practitioners' Column.

[Contributions to this Column are invited, and if accepted will be paid for.]

### POINTS IN PRESCRIBING.

By D. M. MACDONALD, M.D., Dunkeld.

There is a growing danger that prescription writing will become a lost art. The causes need not be discussed at length, but two of the leading ones may with profit be indicated. One is the advance and increase of the products of pharmacy in a portable and palatable form. Instead of committing posological tables to memory, the practitioner has only to remember a number, or, failing that, a name such as Pil. Blaud Co. (Smith and Co.). This is a matter for regret. The mere fact of having the doses of at least the more potent drugs at one's finger-ends is in itself good mental exercise, and if the effort has not to be made, the acquired function soon ceases to exist, while the composition of pill and mixture calls for a knowledge of the action of one drug upon another, and of both upon the body. It is surprising how few medical men there are who originate a prescription of their own, and do not borrow or copy formulæ.

### The Teaching of Pharmacy Neglected.

Another contributing cause is the scanty treatment of practical pharmacy at schools of medicine. Owing to the "hypertrophy of anatomy," a subject of much greater use to the student is almost crowded out of his curriculum. In olden days some of our foremost practitioners spent a portion of their time with a good pharmacist, getting practical knowledge of making up preparations and prescriptions, and so laying the foundation of confident and accurate prescribing in after-life.

### Some Common Mistakes in Prescribing.

It is the purpose of this article to mention some common mistakes which should not be made by any practitioner with a sound knowledge of his work. In the first instance, the prescriber should be precise. How frequently one meets with the following in a prescription:—Aq. menth., liq. morph., hydrarg chlor., bismuth, wherein the intention of the doctor has to be interpreted by the chemist, who may never have seen or heard of the writer of the prescription. It might be argued that liq. morph. always means liq. morphinæ hydroch.; still, in combination with liq. plumbi subacet., for example, precipitation results if the hydrochlorate solution is used.

There are two official solutions of arsenic, an alkaline—Fowler's—and an acid, to suit convenience in prescribing. Fowler's solution is not infrequently prescribed with liq. hydrarg. perchlor. If the pharmacist does not interfere and give the acid solution, the patient may get all the precipitated mercury in the last dose. A similar fate awaits the strychnine if liq. strych. and liq. arsenicalis are given together. The rule, of course, is obvious—namely, to order the acid preparation when the alkaline one induces chemical change. Before leaving strychnine solution one may remark that frequently in heart lesions it is combined with iodide of potassium. This is a mistake, as the alkaloid is in danger of precipitation by iodides.

Iodides, as is well known, set up in some patients all the symptoms of a cold in the head, even in small doses. Changes in the mouth, such as enlargement of the tongue, dribbling of saliva, etc., are occasional unpleasant symptoms. Difficulties in this way may frequently be overcome by giving small doses of arsenic simultaneously.

These salts are in the majority of cases best given alone. A few samples will indicate why.

| | | |
|---|---|---|
| ℞ | Potass. iodidi ... ... ... ... | ʒj. |
| | Spt. ether. nitrosi ... ... ... ... | ʒiv. |
| | Syr. aurantii.... ... ... ... ... | ʒiv. |
| | Aq ad ... ... ... ... ... ... | ℥vj. |
| Ft. mist. | | |

Unless the chemist interferes, and alkalises the normal acid spt. ether. nit., iodine is liberated and the mixture becomes an impossibility.

Again, take the following:—

| | | |
|---|---|---|
| ℞ | Syr. ferri iodidi ... ... ... ... | ʒiv. |
| | Spt. ether nitrosi ... ... ... ... | ʒij. |
| | Glycerini ... ... ... ... ... | ʒiv. |
| | Aq. ad ... ... ... ... ... ... | ℥iv. |

Here even the above artifice is of no avail, since if the spt. ether. nit. is made alkaline precipitation of the iron follows. The mixture is a hopeless one, and should never have been ordered.

It is equally reprehensible to associate iodides with tinct. ferri perchlor., as sometimes occurs, an almost dangerous combination resulting. Iodides are frequently administered with quinine, and if the latter salt be a neutral one it is quite all right. The following prescription is a mistake:—

| | | |
|---|---|---|
| ℞ | Quin. sulph. ... ... ... ... | grs. xxiv. |
| | Acid. nitric. dil. ... ... ... | ʒj. |
| | Potass. iodidi ... ... ... ... | ʒij. |
| | Aq. ad ... ... ... ... ... | ℥vj |
| Ft. mist. | | |

The nitric acid liberates the iodine, which in turn precipitates the quinine. If the dispenser reduces the nitric acid to a few minims, a clear mixture will result.

Consider, too, this favourite formula:—

| | | |
|---|---|---|
| ℞ | Bismuth. subnit. ... ... ... ... | ʒiij. |
| | Sodii bicarb. ... ... ... ... | ʒiij. |
| | Glycerini ... ... ... ... ... | ʒvj. |
| | Aq. ad ... ... ... ... ... | ℥viij. |
| M. ft. mist. | | |

Some pharmacists dispense this by adding boiling water to the two powders, when carbon dioxide is evolved immediately and completely. If dispensed with cold water there is danger of an explosion occurring from the gas being confined in the bottle. It would simplify matters if prescribers would write carbonate of bismuth instead of subnitrate.

There are instances, of course, when decomposition is not only intentional, but distinctly efficacious. For example, the mist. ferri co. B.P. contemplates subsequent formation of ferrous carbonate, as also does the well-known Blaud's pill.

A combination called gargarisma chlorinæ, which, properly prepared, is one of the most efficacious remedies we have in the treatment of a septic throat. The colour, unfortunately, varies with the dispenser, but if dispensed well it should be quite green. The composition is as follows:—

| | |
|---|---|
| ℞ Potass chloratis ... ... ... | grs. xv. |
| Acid. hydroch. ... ... ... ... | ʒs. |
| Aq. ad ... ... ... ... ... | ℥vj. |
| Ft. gargarisma. | |

The secret lies in using crystals of the salt, strong acid, and a dry bottle. After decomposition is complete the water is added in small quantities at a time.

Amongst the drugs in daily use salicylate of soda occupies a foremost place. This salt varies a good deal, both chemically and physiologically.

Take the following very common prescription:—

| | |
|---|---|
| ℞ Sodii salicylatis ... ... ... ... | ʒij. |
| Spt. amm. arom. ... ... ... ... | ʒiss. |
| Tinct. nucis vomicæ ... ... ... | ʒs. |
| Aq. ad ... ... ... ... ... | ℥vj. |
| M. ft. mist. $\frac{1}{6}$ part for a dose. | |

This mixture is quite clear when first dispensed, but in a day or two becomes brown or even black, due to oxidation changes in the salicylate by the ammonia. I have tried substituting potass. bicarb. in the place of the ammonium carbonate. The change occurring is much less marked, and takes a longer time. Here it is important for the medical man to know what is taking place, so as to be able to satisfy any inquiry on the part of the patient as to the cause of the change. One should also avoid combining salicylates with iron preparations. If alone, salicylate of iron, of a reddish colour, is produced; but this may give rise to varying hues depending on the other constituents of the mixture if the salicylate and iron are associated with an infusion, etc.

Salicylates should not be combined with mineral acids, or salicylic acid is precipitated. They are also incompatible with caffeine, with which they are frequently given. For example:—

| | |
|---|---|
| ℞ Sodii salicylatis ... ... ... ... | ʒij. |
| Caffein citratis ... ... ... ... | ʒj. |
| Aq. cinnam. ad ... ... ... ... | ℥vj. |

Here a dense precipitate forms on the top of the mixture. The prescriber may escape the difficulty by adding a little spt. ammon. arom. or by giving the combination in cachet instead of mixture form. It has been pointed out lately that the epistaxis so frequent in influenza is due to administration of salicylate of soda. It would be interesting to know if this occurs with the physiologically pure drug.

